# Supplementary material for: Splice-Junction-Based Mapping of Alternative Isoforms in the Human Proteome
Source: Cell Rep. Author manuscript; Available in PMC 2020 Jan 15. (PMC6961840; doi:10.1016/j.celrep.2019.11.026)
Supplement: 2 [file NIHMS1546469-supplement-2.zip › DF1/PXD006675/RightAtrium/RightAtrium_40_TTN_SSCTAVVDVSEPPSFVK.pdf]

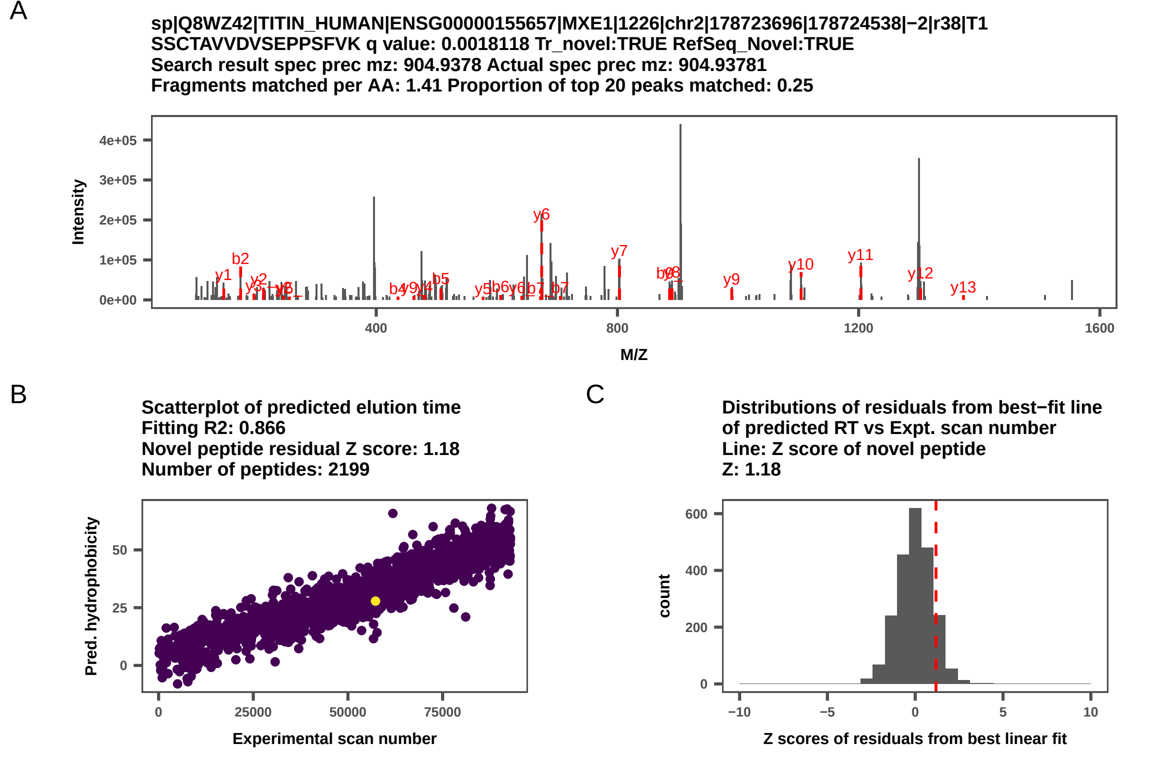
A
sp|Q8WZ42|TITIN_HUMAN|ENSG00000155657|MXE1|1226|chr2|178723696|178724538|−2|r38|T1
SSCTAVVDVSEPPSFVK q value: 0.0018118 Tr_novel:TRUE RefSeq_Novel:TRUE
Search result spec prec mz: 904.9378 Actual spec prec mz: 904.93781
Fragments matched per AA: 1.41 Proportion of top 20 peaks matched: 0.25
Intensity
4e+05
3e+05
2e+05
1e+05
0e+00
M/Z
400
800
1200
1600
y1
b2
y2
y6
y7
y9
y10
y11
y12
y13
b5
B
Scatterplot of predicted elution time
Fitting R2: 0.866
Novel peptide residual Z score: 1.18
Number of peptides: 2199
Pred. hydrophobicity
50
25
0
0
25000
50000
75000
Experimental scan number
C
Distributions of residuals from best−fit line
of predicted RT vs Expt. scan number
Line: Z score of novel peptide
Z: 1.18
count
600
400
200
0
−10
−5
0
5
10
Z scores of residuals from best linear fit
